# Progressive Cognitive Deficit, Motor Impairment and Striatal Pathology in a Transgenic Huntington Disease Monkey Model from Infancy to Adulthood

**DOI:** 10.1371/journal.pone.0122335

**Published:** 2015-05-12

**Authors:** Anthony W. S. Chan, Jie Jiang, Yiju Chen, Chunxia Li, Melinda S. Prucha, Yijuan Hu, Tim Chi, Sean Moran, Tayeb Rahim, Shihua Li, Xiaojiang Li, Stuart M. Zola, Claudia M. Testa, Hui Mao, Rosa Villalba, Yoland Smith, Xiaodong Zhang, Jocelyne Bachevalier

**Affiliations:** 1 Department of Human Genetics, Emory University School of Medicine, Atlanta, Georgia, United States of America; 2 Division of Neuropharmacology and Neurologic Diseases, Yerkes National Primate Research Center, Emory University, Atlanta, Georgia, United States of America; 3 Yerkes Imaging Center, Yerkes National Primate Research Center, Emory University, Atlanta, Georgia, United States of America; 4 Department of Biostatistics and Bioinformatics, Emory University School of Public Health, Atlanta, Georgia, United States of America; 5 Department of Psychiatry and Behavioral Sciences, Emory University School of Medicine, Atlanta, Georgia, United States of America; 6 Department of Neurology and Parkinson’s and Movement Disorders Center, Virginia Commonwealth University, Richmond, Virginia, United States of America; 7 Department of Radiology and Imaging Sciences, Emory University School of Medicine, Atlanta, Georgia, United States of America; 8 Department of Neurology, Emory University School of Medicine, Atlanta, Georgia, United States of America; 9 Department of Psychology, Emory University School of Medicine, Atlanta, Georgia, United States of America; 10 Division of Developmental and Cognitive Neuroscience, Yerkes National Primate Center, Emory University, Atlanta, Georgia, United States of America; Inserm U837, FRANCE

## Abstract

One of the roadblocks to developing effective therapeutics for Huntington disease (HD) is the lack of animal models that develop progressive clinical traits comparable to those seen in patients. Here we report a longitudinal study that encompasses cognitive and motor assessment, and neuroimaging of a group of transgenic HD and control monkeys from infancy to adulthood. Along with progressive cognitive and motor impairment, neuroimaging revealed a progressive reduction in striatal volume. Magnetic resonance spectroscopy at 48 months of age revealed a decrease of N-acetylaspartate (NAA), further suggesting neuronal damage/loss in the striatum. Postmortem neuropathological analyses revealed significant neuronal loss in the striatum. Our results indicate that HD monkeys share similar disease patterns with HD patients, making them potentially suitable as a preclinical HD animal model.

## Introduction

Huntington disease (HD) is a devastating autosomal dominant neurodegenerative disease caused by the expansion of polyglutamine (polyQ) residues (>36Q) in exon 1 of the huntingtin (*HTT* or *IT15*) gene. A major challenge in the study of HD pathogenesis and the development of effective therapies is that the currently available animal models cannot replicate the full spectrum of HD clinical and pathological features, among them motor symptoms, cognitive behavioral deficits, and neuropathological changes of the striatum [1,2,3,4]. Establishing a transgenic monkey model for HD (HD monkeys) could allow us to evaluate the long-term disease process via clinical assessments comparable to those used in human patients and to monitor the effects of novel therapeutics throughout treatment [3,4,5].

There have been many advances in our understanding of HD etiology and pathogenesis. Current research initiatives involve deciphering the mechanisms behind disease onset and improving animal models to aid in the search for a cure. Amongst the key clinical signs of HD are a progressive impairment in motor function and abnormal involuntary movements, such as chorea and dystonia. Recent longitudinal neuroimaging studies of HD patients suggested that non-invasive MRI provides an optimal quantitative tool for determining anatomical changes that are highly correlated with the development and progression of cognitive behavioral and motor impairments throughout the course of HD [6,7,8,9].

We established a group of transgenic HD rhesus monkeys (n = 4; rHD1, rHD6, rHD7, and rHD8; males) and compared them with a group of age-matched non-transgenic control rhesus monkeys (WT monkeys; n = 4; 2 males and 2 females). These animals were enrolled in a longitudinal study that encompassed cognitive and motor assessment combined with *in vivo* neuroimaging and postmortem pathological analyses. The huntingtin (*HTT*) gene of rhesus macaques normally carries 10–11 polyQ repeats [10], while humans normally carry 9–35 repeats [11,12,13]. In our study, rHD1 carried exon 1 of the human *HTT* gene regulated by human polyubiquitin-C promoter, which expressed N-terminal 67 amino acids with 29 polyQ repeats [4]. rHD6, 7, and 8 (rHDs6-8), on the other hand, carried exons 1–10 of the human *HTT* gene coding N-terminal 508 amino acids with approximately 67-72Q under the control of the human *HTT* promoter. rHD1 developed facial chorea and dystonia at around 18 months of age and had his first episode of seizures at 24 months of age. On the other hand, we saw no chorea in rHDs6-8, and dystonia was first observed at 24 months of age (S1 Fig). Because of the distinct genotypes of rHD1 versus rHDs-6-8, a more aggressive form of HD reminiscent of juvenile HD was expected in rHD1 because he expressed a smaller N-terminal mHTT under the ubiquitin promoter [3]. On the other hand, rHDs6-8 were expected to express the mutant *HTT* transgene similarly to the endogenous *HTT* gene. As a result of the regulation by human *HTT* promoter and a larger *HTT* fragment, we anticipated a slower HD progression similar to adult-form HD in rHDs6-8. Our longitudinal characterization of clinically manifested symptoms and neuroanatomical changes in HD monkeys could give us valuable information about the onset and/or progression of HD symptoms and pathology in these animals. The development and evaluation of such a potential preclinical nonhuman primate HD model could be pivotal for the development of novel therapeutics and clinical interventions for the treatment of HD in humans.

## Materials and Methods

### Ethics statement

All animal procedures (e.g., housing, testing) were performed in a BSL-2 facility at the Yerkes National Primate Research Center (YNPRC) and were approved by the Emory University Institutional Animal Care and Use Committee (IACUC). Animal research was conducted in compliance with the Animal Welfare Act and other Federal statutes and regulations relating to animals and experiments involving animals and adheres to the principles stated in the *Guide for the Care and Use of Laboratory Animals* prepared by the National Research Council. Yerkes National Primate Research Center is a fully AAALAC-accredited facility. All animals in the colony are managed in accordance with the applicable USDA Animal Welfare Regulations and the Guide for the Care and Use of Laboratory Animals. All procedures were approved by the Emory Health and Biosafety Committee (EHSO). Transgenic HD monkeys were created by lentiviral transfection of rhesus mature oocytes, followed by *in vitro* fertilization, culture, and embryo transfer into surrogate female macaques as previously described [4]. Cognitive motor assessment was performed and monitored by the research staff and veterinary staff during experimental procedures and on a routine basis following established procedures. All MR imaging experiments were performed using a 3T scanner.

### Production of transgenic Huntington disease monkeys (HD monkeys)

Four male transgenic HD rhesus macaques (“rHD1” and “rHD6, rHD7, rHD8”) were created by transfection of mature oocytes by using a lentiviral vector carrying a mutant form of the *HTT* gene [4]. For rHD1, the insert contained exon 1 of the human *HTT* gene bearing expanded polyglutamine (PolyQ) repeats (29Q and 83Q, respectively) under the control of a human polyubiquitin C promoter, as described previously [4]. For the remaining 3 transgenic HD monkeys, rHDs6-8, the lentiviral vector contained the first 11 exons of the human *HTT* gene bearing the first 508 amino acids and an expanded polyQ (67Q, 70Q, and 72Q, respectively) under regulation by the human *HTT* promoter [5]. All HD monkeys, as well as 4 age-matched wild-type non-transgenic (WT) control monkeys (2 males and 2 females), were raised in the same primate nursery. They all received the same treatments and procedures designed for the longitudinal study, including cognitive motor assessment and MRI scans. All animals were monitored at least twice daily from birth by the research team or by animal care personnel at the YNPRC. In addition to euthanasia guidelines of the YNPRC, we have developed specific euthanasia guidelines based on functional assessment of HD monkeys; we assess feeding behavior, monitor weight, urination, and defecation, and daily activity, as well, as if the animals are alert and responsive. Besides daily functional assessment, the occurrence of adverse events, such as the development of self-injurious behavior (SIB), prompts us to seek an immediate veterinarian consultation for appropriate resolution and medications. Euthanasia is considered if medical justification is reached. In the case of rHD1, SIB developed initially due to stress, such as during the cognitive behavioral test (e.g., Life Saver Task). Our first step to minimize the suffering of the animal from abnormal behavioral conditions was to stop all tests to eliminate the potential stress. Due to the progression of the disease and continued development of SIB, medications, including analgesics, anticonvulsants, and antidepressants, were provided to relieve the suffering of rHD1. However, euthanasia had to be considered based on concerns for the well-being and medical condition of the animal.

Post-delivery, infants were surrogate-nursery reared in the primate nursery of the YNPRC according to procedures developed by Sackett and colleagues [38] that allow normal growth as well as the development of species-specific social skills. These procedures included daily social interactions with peers, intensive human contact, and cognitive testing that began in the first weeks of life and continued through adulthood [39].

Diet consisted of infant Similac formula (SMA with iron) supplemented with banana pellets starting at 3–4 weeks old (190 mg, P.J. Noyes, Cleveland, OH). Starting around 8 months of age, they were fed jumbo primate chow (Lab Diet #5037, PMI Nutrition International Inc., Brentwood, MO) and fresh fruit daily. All experimental procedures were approved by the Institutional Animal Care and Use Committee of Emory University (Atlanta, GA) and conformed to the NIH Guide for the Care and Use of Laboratory Animals.

### Behavioral testing

HD and WT monkeys were tested at different points throughout development to follow their neurobehavioral and motor development. Animals were transferred to a Wisconsin General Testing Apparatus (WGTA), facing a test tray onto which objects or equipment could be positioned. Rewards were given according to each animal’s preference, and included either peanuts, raisins, fruity gems, mini M&M’s, marshmallows, or a ring-shaped candy (“Life Saver”) for the VS-OR task (see below).

For the Object Retrieval Detour Task (ORDT; Detour/Barrier), the apparatus consisted of a small transparent box (5” x 5” x 4”) opened on only one side and fixed on a tray. The box could be located at different positions on the tray and could be rotated so that its open side could be changed. The positions of the reward within the box (at the entrance but out of the box, one-third in, half-way in, and three-quarters in), the box on the tray (left, right, center), and the orientation of the open side of the box relative to the subject (front, left, right) were parametrically varied. Subjects were allowed unlimited time to retrieve the reward as long as they continued attempting to respond. A maximum of 3 minutes during which no responses occurred terminated the trial and was scored as a “failure.” Training consisted of 7 testing days as follows. Day 1: Fifteen trials were given with the opening of the box facing towards the animal, such that all trials involved a direct reach for the reward not requiring the animal to inhibit reaching at the barrier. Days 2–4: Test sessions consisted of 18 trials, in which the trials required the animal to either make a direct reach (box opening facing the animal) or to negotiate a detour (box opening facing right or left of the animal). These 2 trial types were intermixed within a session. Days 5–7: The plastic box was positioned at the center of the box for all trials. Daily test sessions consisted of 21 trials requiring the animal to negotiate a detour on one side 5 times in the row, followed by a switch requiring the animal to negotiate a detour in the other side 5 times in a row and so on (e.g., box opening on the left, left, left, left, left, then box opening on the right, right, right, right, right, etc.). These trials were included to increase the tendency for animals to exhibit perseverative responses, defined as successive repetitions of a previously rewarded reach. All testing was video recorded, and the video tapes were subsequently analyzed by an experimenter blind to animal treatment (HD or WT). Several measures of performance were included and are listed in S1 Table. Animals were tested on this task at 2 developmental time points (8 and 16 months).

In the visuospatial orientation (VS-OR) task, the monkeys had to free a ring-shaped candy (“Life Saver”) by moving it along a metal rod that was bent into simple to complex detour patterns or routes. A testing tray with a vise at its center onto which metal-rods were secured was used. Metal rods were either straight for the pre-training phase or had 1–3 bends for the “easy routes” and 4–5 bends for the “difficult routes” (see Fig 1 in Bachevalier et al., 1991 for details of the metal rods used) [22]. On each trial the central pole of a metal-rod route was inserted into the vise, and a Life Saver was threaded along the rod back to the starting point, located on either the right or the left side of the central pole. Monkeys were required to retrieve the Life Saver by threading it to the free end of the rod. The test was composed of 3 stages. Pre-training stage: the animal learned to remove the Life Saver from a straight rod placed in 1 of 4 different orientations (up, right, left, and toward the monkey) during 12 daily sessions. The monkey was given 6 practice trials for each orientation in which the experimenter helped the animal free the Life Saver if necessary. Six test trials at each orientation on each of 3 days were performed after the practice trials. The monkey performed these test trials without experimenter help, and the time to retrieve the Life Saver was recorded. Second stage: pseudorandomly 3 times each for a total of 36 trials were presented as 12 “easy” routes. Each animal received 6 of these trials per day for 6 consecutive days, and a maximum of 45 seconds was allowed to retrieve the Life Saver. For the third and final stage, 12 “difficult” routes were presented similar to the second stage, and a maximum of 120 seconds were allowed to retrieve the reward. This task was given at both 16 and 36 months, except for rHD1, who was tested at the first time point only (i.e. 16 months). The VS-OR task or the “Life Saver” task procedure was previously described in detail by Bachevalier and colleagues in 1991 [22].

**Fig 1:**
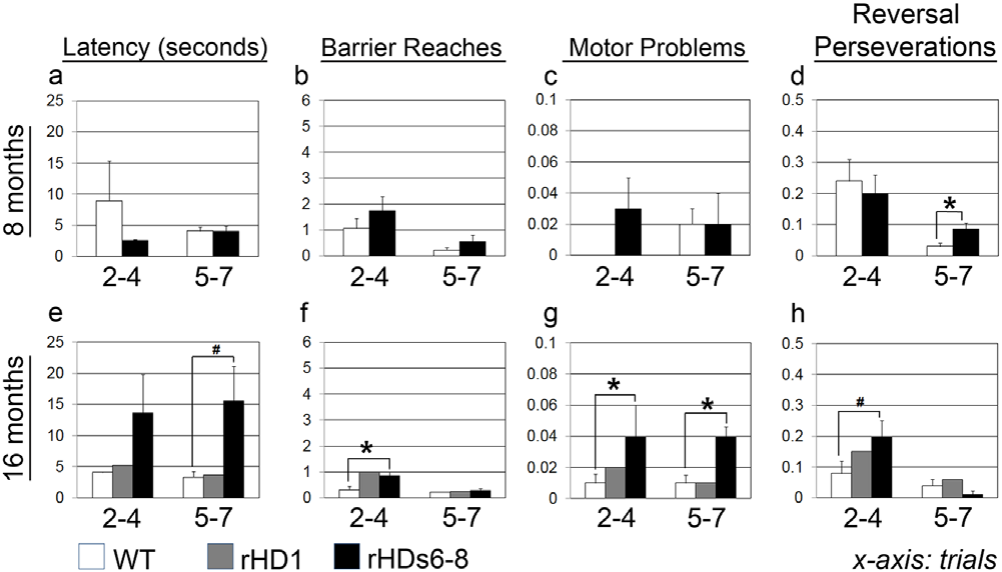
Object retrieval detour task (ORDT) at 8 and 16 months of age. ORDT was performed at 8 (a-d; top panel) and 16 (e-h; bottom panel) months of age. Scores are (a, e) latency (sec) to retrieve the reward, (b, f) frequency of reaching through the transparent plastic barrier, (c, g) number of motor problems, and (d, h) frequency of reaching repetitively through the transparent wall opposite to the opening. Data were collected from HD and WT monkeys (n = 4 per group). # indicates near significance (p = 0.07) and * indicates a p-value < 0.05.

Statistical comparisons for all behavioral data were carried out using the SYSTAT 13 software. Comparisons between controls and HD animals were performed using 2-way ANOVA with repeated measures when the assessments were done longitudinally across ages, followed by post-hoc analyses. One-sided, 2-sample t-tests were used to conduct between-group comparisons. When data were not distributed normally, Mann-Whitney U tests were used for group comparisons.

### MR image and spectroscopy data acquisition

All MRI experiments were performed using a Siemens 3T Trio scanner (Siemens Medical Solutions, Inc, Malvern, PA, USA) with a CP extremity volume coil (for 6-, 12-, 18-, and 24-month scans) and an 8-channel phased-array knee coil (Invivo Inc, FL, USA) (for 30-, 36-, 42-, and 48-month scans). Animals were immobilized in the supine position with the head secured in a custom-made head holder. Anesthesia was maintained with 1–1.5% isoflurane mixed with O_2_. Et-CO_2_, inhaled CO_2_, O_2_ saturation, blood pressure, heart rate, respiration rate, and body temperature were monitored continuously and maintained in normal ranges [40]. T1-weighted images were acquired using a 3D magnetization-prepared rapid acquisition gradient echo (MPRAGE) sequence with the parameters: TR = 2500 ms, TE = 3.48 ms, TI = 950 ms, FOV = 96 mm x 96 mm, data matrix = 192 X 192, flip angle = 8 degree, slice thickness = 0.5 mm, 208 slices, and 6 averages. Single-voxel proton magnetic resonance spectroscopy (^1^H-MRS) was performed using a PRESS (Point Resolved Spectroscopy) sequence combined with the CHESS water suppression with TE = 30 ms, TR = 1500 ms, and voxel size = 5 × 5 × 5 mm^3^. The cubic voxel was placed in the right striatum and illustrated on 3 orthogonal structural images.

#### Volume calculation for whole brain and cortical structures

In order to calculate the total brain volume (TBV), the skulls were stripped from all images using the FSL Brain Extraction Tool (BET) [41]. Skull-stripped brain images were then used to calculate the TBV. Each structure (caudate nucleus and putamen) was manually outlined on coronal T1-weighted images using a monkey brain atlas [42]). The volume of each structure was then calculated with ImageJ 1.47v (NIH). The striatal volume is the summation of caudate nucleus and putamen.

#### Magnetic resonance spectroscopic (MRS) analysis of NAA in the striatum

Concentrations of cerebral metabolites, including N-acetylaspartate (NAA), creatine, and phosphocreatine (Cr+PCr), were derived from the spectra of each animal at 48 months of age using the LC-Model software [43]. Total creatine (Cr+PCr) levels were used as an internal reference for the quantification of other metabolites [44,45]. The spectral results were analyzed and compared statistically among the disease and control groups using Student’s *t-test*.

#### Statistical analysis of longitudinal MRI data

Linear mixed-effects models, carried out by the “lme” function in R (www.r-project.org) version 2.15.1, were fitted to the longitudinal data to account for the correlation of repeated measurements from the same subject. Specifically, we used the model:

$$Y_{it}\;=\;\beta_{0}+\;\beta_{1}HD_{i}\;+\;\beta_{2}time_{t}\;+\;\beta_{3}\times HD_{i}\times time_{t}\;+\;\beta_{\text{4}}time_{t}{}^{2}\;+\;\beta_{5}\times HDi\times time_{t}{}^{2}\;+\;bi\;+\;\varepsilon it$$

where *Y*_*it*_ is brain volume measurement at any ROI for the i^th^ subject at the t^th^ time point, *HD*_*i*_ is 1 for HD monkeys and 0 for controls, *time*_*t*_ is month of the t^th^ time point, *bi* is a random effect that follows N(0, $\sigma_{b^{2}}$), and *ε*_*it*_ is an error term that follows N(0, *σ* ^2^) and is independent of *b*_*i*_. To test for any difference in the trajectories between the HD and control monkeys, i.e., H0: *β*_*1*_ = *β*_*3*_ = *β*_*5*_ = 0, we conducted the likelihood ratio test (LRT). P-values less than 0.05 were considered statistically significant.

### Reverse transcription and quantitative PCR (qPCR)

Total RNA from the caudate nucleus and putamen was isolated using Trizol and subsequently treated with DNase to remove residual genomic DNA contamination. 500 ng DNA-free total RNA was reverse transcribed in a total volume of 20 μL using MultiScribe Reverse Transcriptase (Applied Biosystems) as outlined in the manufacturer’s protocol. Primers used for real-time PCR were designed using Primer3Express and are listed below. Each qPCR reaction consisted of 1X SsoAdvanced SYBR Green Supermix (Bio-Rad) with 0.4 μM each primer (S2 Table), and thermal cycling was performed according to the manufacturer’s protocol. All *HTT* exon 1 data (primers amplified endogenous and transgenic *HTT*) were analyzed by normalization to both ubiquitin C (*Ubc*; housekeeping gene) and/or *HTT* exon 26 (primers specifically amplified endogenous *Htt* only).

### Neuropathological analysis

#### Tissue preparation

Monkeys were deeply anesthetized with an overdose of pentobarbital at the time of sacrifice. Brains were rapidly removed from the skull and post-fixed with 4% paraformaldehyde, and then cryoprotected with 30% sucrose. The brains were frozen-sectioned into serial 50-μm thick coronal sections and preserved at -80°C.

#### NeuN and mEM48 staining

Sections from one control, rHD1, and rHD7 monkeys were immunostained with a specific primary antibody for neuronal nuclei (NeuN) and mHTT with expanded polyQ (mEM48). Sections were then stained with associated secondary antibody and localized with avidin-biotin-peroxidase complex (ABC, VECTASTAIN ABC kit). Diaminobenzidine (DAB) was used for the peroxidase reaction to reveal the staining.

#### Stereological cell counting

One in 12 sections of control and HD monkeys were Nissl-stained with cresyl violet. These sections were used for unbiased stereological cell counting (Stereo Investigator, MBF BIOSCIENCE). The optical fractionator probe was used for counting cell numbers. A region of interest (ROI) of striatum was delineated using a 2.5X objective, and the sections were then examined with a 100X objective. Cells were counted at 100X from 65 x 65 μm unbiased counting frames within a randomly chosen 1200 x 1200 μm square grid. An optical dissector height of 27 μm and a top guard zone height of 3 μm were used. The total number of cells in the ROI was estimated with the section sampling fraction, the area sampling fraction, and the mean section thickness [46].

## Results

### Cognitive and motor assessment of HD monkeys

At 8 and 16 months of age, animals performed the object retrieval detour task (ORDT; Barrier/Detour; S1 Table). Performance at 8 months of age was similar between rHDs6-8 and WT monkeys (Fig 1a–1d; top panel), although the former displayed a slightly higher number of perseverations in the difficult trials (Fig 1d) [t = 3.315 with 5 d.f., p = 0.021] (rHD1 was not assessed at 8 months of age). Notably, testing conducted on HD monkeys at 16 months of age revealed a gradual impairment in motor function (Fig 1e–1h; bottom panel). They took longer to retrieve the rewards than controls in both the easy (Trials 2–4) and difficult trials (Trials 5–7), although this group difference showed only a trend towards significance for the difficult trials [t(5) = 2.34, p = 0.06] (Fig 1e); rHDs6-8 also performed more barrier reaches [t(5) = 2.60, p = 0.048] (Fig 1f) and tended to have more perseverative errors [t(5) = 2.21, p = 0.07] (Fig 1h) in the easy trials. They displayed significantly greater motor problems when attempting to retrieve the food rewards in all trials [U = 0.5, p = 0.04 and U = 0, p = 0.026, for easy and difficult trials, respectively; Fig 1g]. Of note, although difficulties gradually emerged in rHDs6-8 as they matured, rHD1 showed no impairment in the task at 16 months of age.

**Fig 2:**
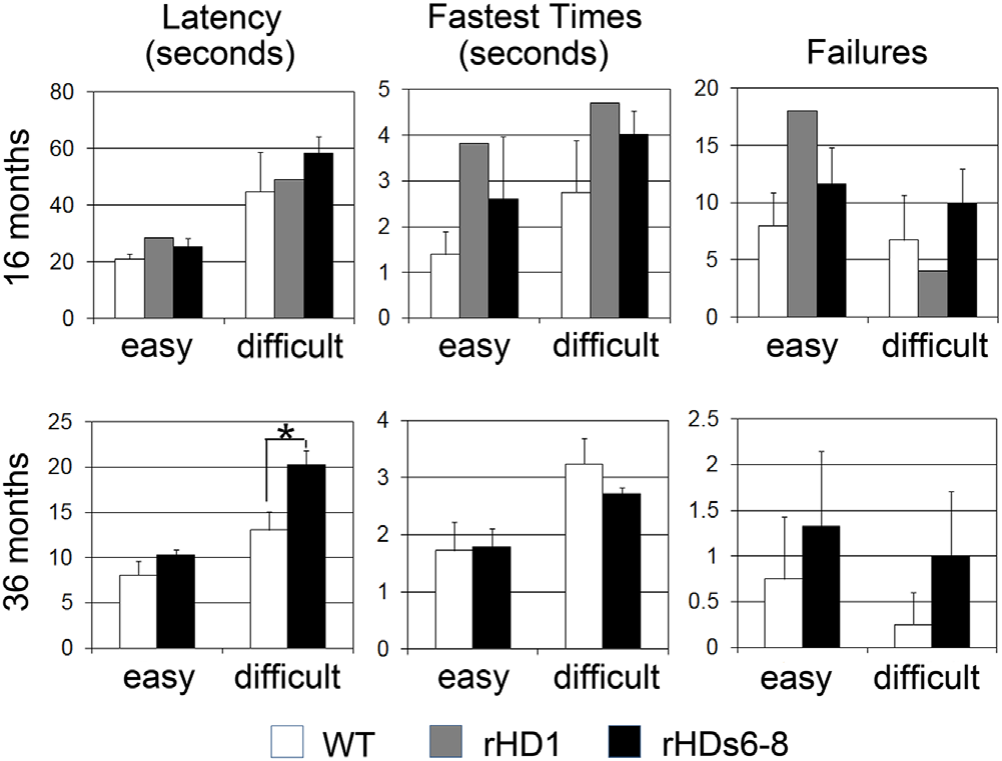
Visuospatial orientation (Life Saver) task at 16 and 36 months of age. Life Saver task was performed at 16 (left panel) and 36 (right panel) months of age. Latency (sec) and fastest times (sec) to free a ring-shaped candy (“Life Saver”) by moving it along metal rods of increasing difficulty. Easy rods contained 1–3 bends, and difficult rods contained 4–5 bends. Please note that testing of rHD1 in the Life Saver task had to be discontinued due to the development of self-injurious behavior during testing. * indicates a significant difference between HD and WT monkeys (p < 0.05).

Visuomotor performance was assessed further at both 16 and 36 months of age using the visuospatial orientation (VS-OR; Life Saver) task. At 16 months of age, there were no significant differences between the HD and WT monkeys in any of the parameters tested (latency, fastest times, and failures; Fig 2, left panel). At 36 months of age, however, rHDs6-8 required more time to perform the task when more difficult patterns were used [t(5) = 3.73, p = 0.014, for latency; Fig 2, right panel]. Note that testing of rHD1 in the Life Saver task was discontinued due to the development of self-injurious behavior during the time this test was being administered.

### Volumetric changes in the striatum of HD monkeys

**Fig 3:**
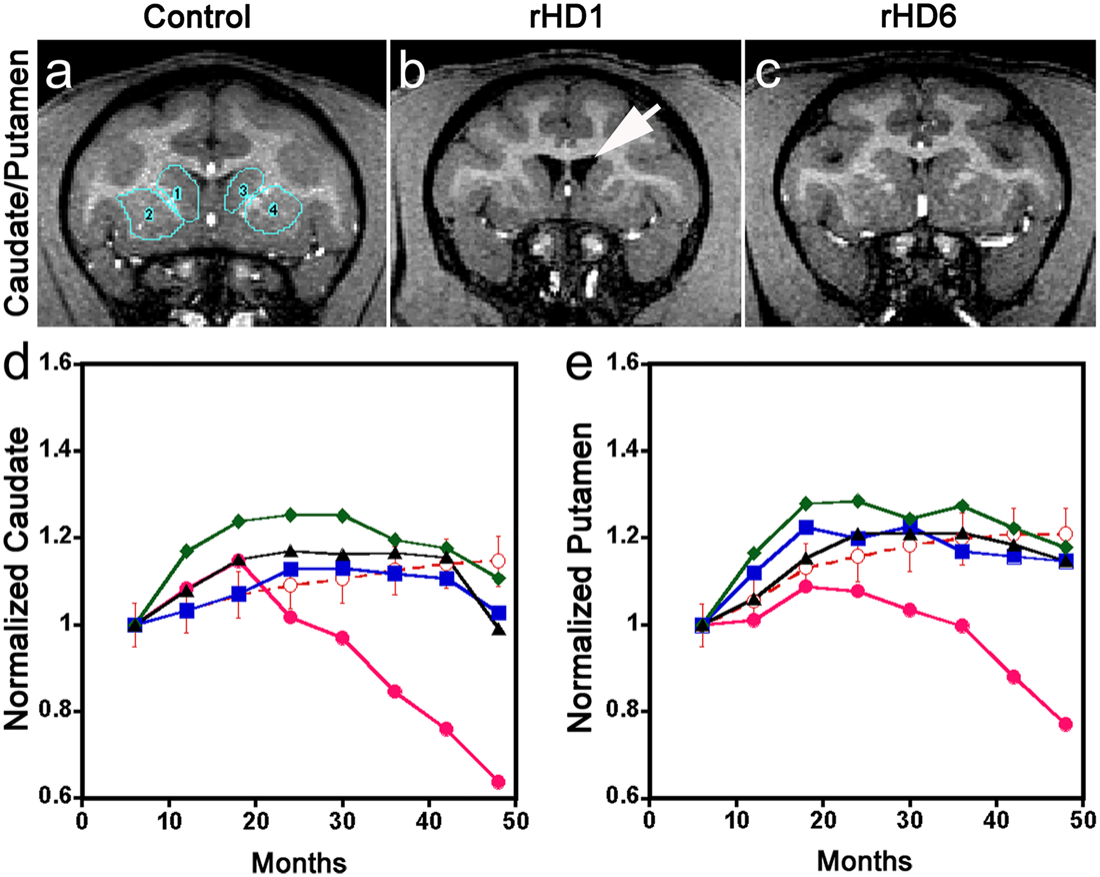
Longitudinal changes of striatal (caudate/putamen) volumes in HD monkeys. (a-c) Representative T1-weighted images of a control monkey, rHD1, and rHD6 indicating the regions of interest of the caudate nucleus and putamen. (d) Longitudinal changes in caudate nucleus. (e) Longitudinal changes in putamen. Control (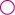), rHD1 (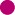), rHD6 (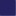), rHD7 (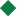), and rHD8 (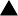). Arrows indicate enlarged ventricles.

We performed 4 years of semiannual longitudinal measurements of volumetric changes in the caudate nucleus and putamen using brain MRI data and normalized to the total brain volume (TBV) for each individual in order to minimize inter-subject variations (Fig 3). Unlike rHD1, which diverged distinctly from WT at 24 months of age (Fig 3d and 3e), the volumetric reduction of both the caudate nucleus and putamen of rHDs6-8 was not significantly different from control at each time point (Fig 3d and 3e). There was progressive enlargement of the ventricles of rHD1 as the disease progressed, further confirming the occurrence of regional atrophy in the area of the striatum (S2 and S3 Figs). Although no statistically significant striatal volumetric reduction was observed in rHDs6-8 at specific ages (Fig 3d and 3e) when compared with WT monkeys, we did see significantly different brain volume expansion trajectories (p < 0.05) as determined by the likelihood ratio test (LRT). The overall trajectory of volumetric changes in rHDs6-8 over the period of this study indicated a decreasing inclination, which was significantly different from the WT monkeys. The brain volumes of rHDs6-8 stopped increasing by 24 (rHD7) or 36 (rHD6, 8) months of age, whereas brain volumes in WT monkeys continued to grow at a slow and steady rate until the end of the 4-year study as they approached adulthood (Fig 3d and 3e).

In parallel with the brain volume measurement using neuroimaging data, we measured head size and body weight every 6 months to determine whether HD monkeys had different growth trajectories (S4 Fig). After adjusting for gender, both the head size and body weight of HD monkeys demonstrated significantly different trajectories compared with WT monkeys (p < 0.0002 and p < 0.0001, respectively). To further confirm that differences in brain volume were not due to head size or body weight, brain volume was adjusted for head circumference, body weight, and gender by including these parameters as covariates in the statistical analyses. The trajectories of brain volume in different regions between HD and WT monkeys remained significantly different, with high significance (p < 0.0001).

### Assessment of mHTT transgene expression, formation of HTT aggregates, and neural cell loss in caudate nucleus and putamen

**Fig 4:**
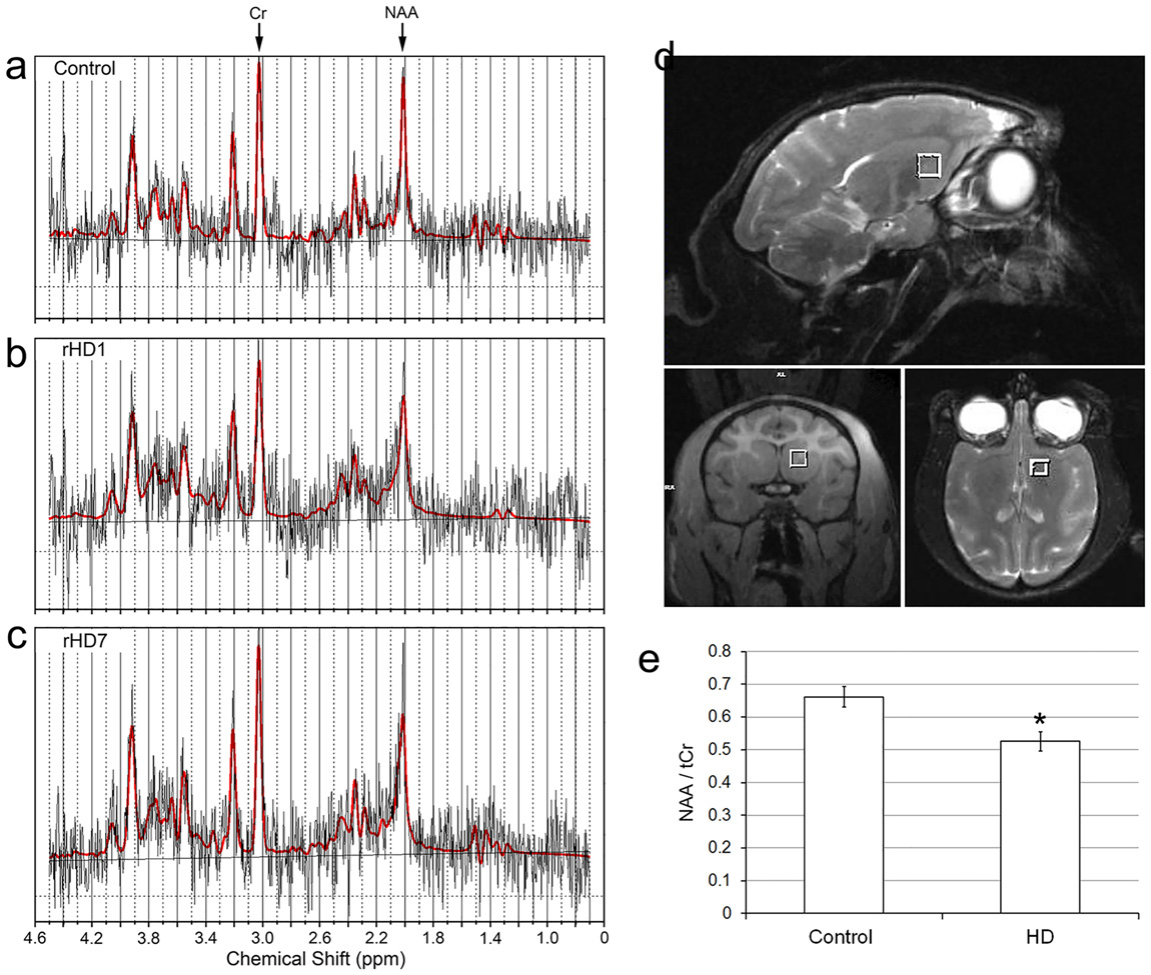
Measurement of NAA levels in the striatum of HD monkeys at 48 months of age by proton magnetic resonance spectroscopy (^1^H MRS). ^1^H MRS obtained from (a) Control, (b) rHD1, (c) rHD7, showing peaks of creatine (Cr) and N-acetylaspartate (NAA). (d) Location of the voxel used for ^1^H MRS in the striatal region (white squares). (e) Comparison of NAA level between control (n = 4) and HD monkeys (n = 4). *p < 0.05.

**Fig 5:**
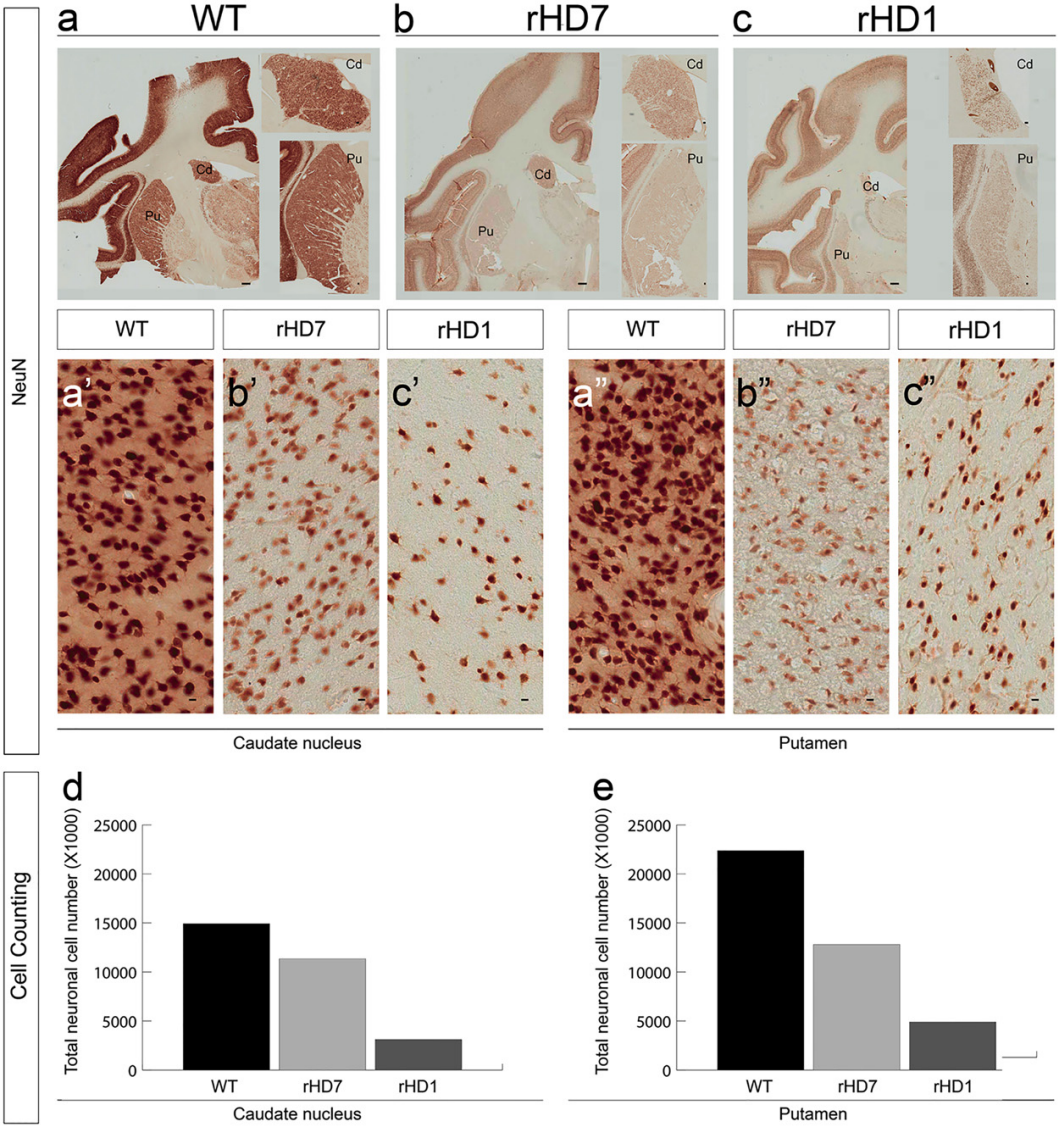
Reduced striatal neurons in the caudate nucleus and putamen of HD monkeys. Immunostaining with the neuronal-specific marker NeuN in the striatum in 5-year-old control (a), rHD7 (b), and rHD1 (c) monkeys (scale bar = 1mm). The close-up view at the right side shows a closer view of the caudate nucleus and putamen (scale bar = 100μm). The number of NeuN-positive neurons in the caudate nucleus (a’, b’, c’) and putamen (a”, b”, c”) of the HD monkeys is dramatically reduced compared with the control animal (scale bar = 10 μm). By counting from the Nissl staining, the number of neurons in the caudate nucleus and putamen is reduced in both HD monkeys when compared to control monkeys (d, e, respectively).

**Fig 6:**
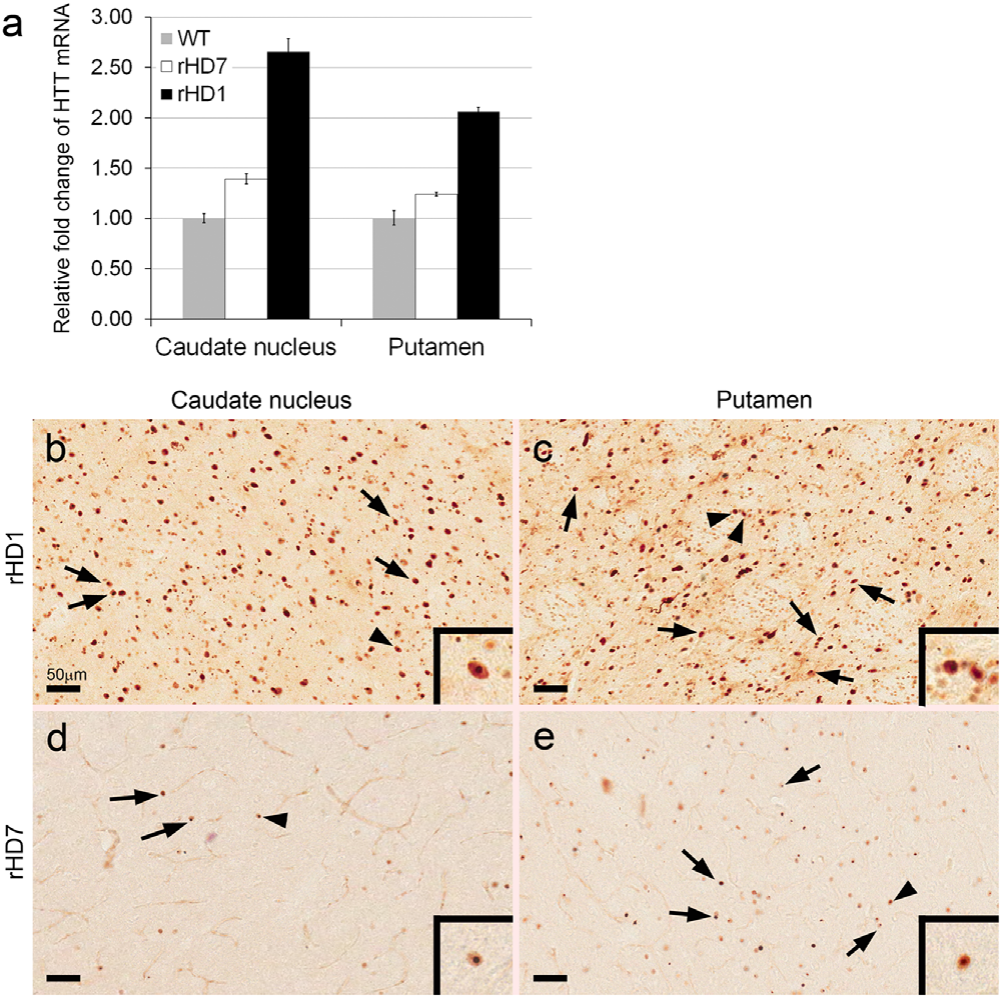
Expression of mHTT transgenes and formation of mHTT aggregates in the caudate nucleus and putamen of HD monkeys. Quantitative measurement of mHTT transcript levels in the caudate nucleus and putamen by qPCR (a). Immunostaining with mEM48 that specifically recognized mHTT with expanded polyQ repeats in the caudate nucleus and putamen of rHD1 (b and c) and rHD7 (d and e), respectively. Scale bar = 50 μm. Arrows: mEM48-positive cells with intranuclear inclusions. Arrowhead: mEM48-positive cell presented at higher magnification in the insert.

Although we saw progressive cognitive and motor impairment in rHDs6-8, volumetric measurement of the caudate nucleus and putamen by MRI suggests no significant changes at each time point, even though significant declining trajectories were seen in the rHDs. To determine whether there was loss or damage to neuronal tissues in the striatum of HD monkeys, *in vivo* proton magnetic resonance spectroscopy (^1^H MRS) was used to measure striatal changes of NAA, a surrogate marker of neuronal integrity, in the striatum (Fig 4a–4d). A decrease of NAA has been used as an indicator for metabolic disturbances and neuronal loss in HD [14,15]. MRS data showed a significantly lower level of NAA in the striatum of HD monkeys at 48 months of age when compared to the control monkeys (Fig 4e). The reduction of the NAA level suggests neuronal damage or loss and was consistent with neural cell loss seen in the caudate nucleus and putamen of rHD1 and rHD7 (Figs 4 and 5). To further examine whether striatal pathological changes could have contributed to the cognitive and motor deficits seen in these animals, a quantitative neuropathological analysis was performed on the brains of 2 HD monkeys (rHD1 and rHD7) when they were 5 years old. The expression levels of *mHTT* transcript in the caudate nucleus and putamen were measured by qPCR (Fig 6a). As expected, rHD1 had the highest *HTT* transcript levels compared to rHD7 and control, while the control monkey had the lowest (Fig 6a). The expression levels of *mHTT* were consistent with the extent of HTT aggregates and intranuclear inclusions as shown by immunostaining on brain sections using mEM48 antibody that specifically recognized mHTT with expanded polyglutamine repeats (Fig 6b–6e)[4]. rHD1 displayed more positively stained cells with intranuclear inclusions (Fig 6b and 6c) compared with rHD7, whose positively stained cells were seen sparsely throughout the sections (Fig 6d and 6e).

There was a significant volume reduction of the caudate nucleus and putamen in rHD1 (Fig 5A and 5C), which was consistent with the MRI study (Fig 3; S2 and S3 Figs). Unlike rHD1, rHD7 displayed no obvious reduction in striatal volume or enlargement of ventricles compared to the control at specific time points (Fig 5a and 5b); however, the total cell count by stereological analysis of the caudate nucleus and putamen of rHD1 and rHD7 revealed a significantly lower number (caudate/putamen: Control-15000/22500; rHD7-11000/12500; rHD1-2500/5000) of Nissl-positive (Fig 5d and 5e) and NeuN-positive cells (Fig 5b’ and 5c’; Fig 5b” and 5c”) when compared to the age-matched control monkey (Fig 5a’ and 5a”). Comparing rHD1 and rHD7, the striatum of rHD1 contained a much lower number of Nissl-positive cells (Fig 5d and 5e).

## Discussion

This report focused on the longitudinal development of a spectrum of clinically and neuropathologically relevant features in HD monkeys from infancy to adulthood; their symptoms included progressive cognitive and motor impairments, and neuropathological and chemical changes of the striatum. To evaluate cognitive and motor performances, we used the ORDT to assess the functional deficit of the fronto-striatal pathway, which is known to be one of the neural circuits affected in HD [16,17,18,19]. This task, which is commonly used in nonhuman primate models of HD [17,18,19] and Parkinson’s disease (PD) to assess changes in motor planning skill, is sensitive for detecting frontal cortex and striatal dysfunction [16,17,18,19,20]. To further assess motor control as well as motor skill learning and reaction time, the “Life Saver” task was used to interrogate the integrity of the mid- and posterior-putamen functions. The Life Saver test has been widely used in nonhuman primate models of HD [21] and PD to assess motor functions and visuospatial orientation [21,22,23,24,25]. Together, the ORDT and Life Saver tasks revealed that rHDs6-8 developed progressive motor dysfunction and a lack of behavioral inhibitory control; both of these symptoms are associated with HD onset/progression in humans and suggest a functional decline of the fronto-striatal pathways in HD monkeys [16]. These findings are consistent with the decreased brain volume in the striatum measured from neuroanatomical imaging and neuronal loss found in neuropathology, and MRS analyses. It is interesting to note that HD monkeys carrying 2 different transgenes (rHD1 vs rHDs6-8) displayed different patterns of cognitive and motor impairment as well as neuroanatomical changes (Figs 1–3, 5, 6 and S1 Fig).

The distinct pattern of transgene expression (Fig 6a), HTT aggregates and intranuclear inclusions (Fig 6b–6e), changes in striatal volume (Fig 3), neural cell loss (Fig 5), and reduced levels of NAA (Fig 4) in rHD1 and rHD7 further point to differences between HD monkeys that carry different transgenes. rHD1 developed dystonia at least 6–8 months earlier than the other rHDs and was also the only HD monkey to develop seizures at around 24 months of age (S1 Fig). An aggressive striatal atrophy in rHD1 began at 24 months of age, with approximately 47% and 30% volume reduction in the caudate nucleus and putamen at 48 months of age, respectively (Fig 3). In contrast, the other 3 HD monkeys exhibited a relatively slower reduction rate and blunted growth of the striatum at 24 and 36 months of age, while WT monkeys continued to grow throughout the period of this study (Fig 3). Although we saw significant declining trajectories in each of rHDs6-8, there was no significant reduction of the caudate nucleus and putamen volume at any specific age based on longitudinal neuroimaging study (Fig 3), which was further confirmed by the neuropathological examination of rHD7 brain (Fig 5). Interestingly, although we saw no obvious striatal atrophy in rHD7 when compared to control and rHD1, who displayed enlarged lateral ventricles and significant striatal volume reduction (Fig 6a–6c), we found a significant loss (~30–50%) in Nissl- and NeuN-positive cells in the caudate nucleus and putamen of rHD7 (Fig 5). Although only one control and two HD monkeys were used for the neuropathological study, the extent of regional atrophy in rHD1 and the differences in the number of Nissl- and NeuN-positive cells between the control and HD monkeys are clear (Fig 5). While there was no obvious atrophy in rHD7 based on both MRI and neuropathology, the significant cell loss in both caudate nucleus and putamen suggests a progressive regional degeneration, which was further supported by the decrease of NAA at 48 months as measured by ^1^H MRS (Fig 4). A similar change in NAA was seen in rHD6 and rHD8, suggesting the presence of possible striatal neurodegeneration even though there was no noticeably significant reduction of caudate nucleus or putamen volume (Figs 3 and 5). The loss of neural cells in the striatum of rHD1 and rHD7 is consistent with our findings of cognitive and motor impairment in these animals, possibly due to dysfunction in the fronto-striatal pathways and abnormal striatal outflow (Figs 1 and 2). Based on our assessments, rHD1 presented with more acute development of symptoms similar to juvenile HD, specifically with several episodes of seizures, more aggressive development of dystonia at a young age and on throughout adulthood, and significant atrophy of the caudate nucleus and putamen [26,27,28,29]. On the other hand, symptoms in rHDs6-8 were relatively mild, sporadic, and progressed at a much slower rate and in a less aggressive manner compared to rHD1. These different behavioral outcomes were expected based on the different genotypes of these animals, with rHDs6-8 mimicking more closely the slow progression of the adult form of HD.

There are ongoing clinical longitudinal studies of HD patients, including TRACK-HD and PREDICT-HD [9,30]. HD monkeys can be assessed based on human longitudinal trial results and offer expanded assessment and therapeutic development opportunities that directly complement the human subject work. Our parallel study using a large nonhuman primate animal model that closely displays the progressive clinical features of HD offers a unique platform for preclinical assessment of new treatments using cognitive behavioral tests of functions and neuroimaging methods for measuring neuroanatomical ad chemical changes. Moreover, rhesus macaques share a motor repertoire similar to humans, allowing the evaluation of fine movement control, which is impossible with most of the currently available model systems [31]. Most importantly, we can evaluate the neurobehavioral impact of disease and treatment with a sophisticated battery of cognitive and behavioral tests developed for macaques, which is often a challenge in other species [21,32,33].

Since there is no perfect animal model for human diseases, an animal model that can replicate human disease genetically and clinically is important to study specific pathogenic mechanisms underlying inherited neurological disorders like HD that progresses as individuals age [31,34]. A higher primate model such as rhesus macaque can play an important role in modeling diseases like HD because various biological systems, including those that regulate cognitive, motor, and psychiatric functions [2,3,4,9,21,30], as well as metabolism [35,36,37], are progressively impacted throughout the course of the disease. Because of its progressive nature, large animal models, such as HD monkeys, could more effectively mimic the progression of HD-like symptoms through longitudinal analysis with clinical measurements similar to those performed in HD patients [3,5]. A direct comparison and alignment of disease symptom progression with the human condition based on clinical features is a challenging task because of the fundamental differences between species. Our goal is to establish a progression timeline for disease development in HD monkeys by integrating information generated from different clinical assessments (S1 Fig). Although this timeline may not align perfectly with humans with HD, and there will be questions as to how well HD monkeys represent humans, we believe our long-term longitudinal studies in HD monkeys thus far represent one of the most extensive and comprehensive animal studies. This nonhuman primate model encompasses progressive development of HD from infancy to adulthood based on clinical assessment methods similar to those used in humans. However, we acknowledge that our sample size in this study is limited and that additional HD monkeys are needed to further confirm our findings and for the future development of the HD monkey model. Nonetheless, longitudinal assessments on other cognitive functions are ongoing and are expected to further support the initial results of the current study that HD monkeys could develop symptoms similar to those of human HD patients.

## Conclusions

We present the findings of progressive decline in cognitive and motor functions and related neuroanatomical and chemical changes in HD monkeys based on longitudinal assessments from infancy to adulthood. The neuropathology in two of the HD monkeys further demonstrated a progressive degenerating process in the striatum of HD monkeys and indicated that extensive cell loss precedes significant loss of brain mass in striatum. Comparison of HD monkeys also suggests that different N-terminal HTT fragments cause distinct neuropathology. This study demonstrates the potential of transgenic HD monkeys to model human HD in facilitating preclinical research and advancing biomedical research in the quest for a cure for HD.

## Supporting Information

S1 Fig Timeline of progressive development of clinical features in HD monkeys. Specific types of impairment, including cognitive motor functions and neuroanatomical changes, are plotted on a timeline for rHDs6-8 (top) and rHD1 (bottom).
(TIF)

S2 Fig Coronal T1-weighted images of WT control monkey and rHD1 at 12, 24, 36, and 48 months of age. (TIF)

S3 Fig Volumetric changes in HD brain observed in T1-weighted images of rHD1 and a control monkey at four years of age. Arrows indicate enlarged ventricle.
(TIF)

S4 Fig Head circumference and body weight of HD monkeys. Controls 1 and 2 are female monkeys. Controls 3 and 4 are male monkeys.
(TIF)

S1 Table Definitions of behavioral measures in the ORDT. (DOCX)

S2 Table List of primers. (DOCX)

## References

[1] Brouillet E, Conde F, Beal MF, Hantraye P. Replicating Huntington's disease phenotype in experimental animals. Prog Neurobiol. 1999; 59: 427–468. 10515664 10.1016/s0301-0082(99)00005-2

[2] Pouladi MA, Morton AJ, Hayden MR. Choosing an animal model for the study of Huntington's disease. Nat Rev Neurosci. 2013; 14: 708–721. 10.1038/nrn3570 24052178

[3] Chan AW, Xu Y, Jiang J, Rahim T, Zhao D, Kocerha J, Chi T, et al A two years longitudinal study of a transgenic Huntington disease monkey. BMC Neurosci. 2014; 15: 36 10.1186/1471-2202-15-36 24581271 PMC4015530

[4] Yang SH, Cheng PH, Banta H, Piotrowska-Nitsche K, Yang JJ, Cheng EC, et al Towards a transgenic model of Huntington's disease in a non-human primate. Nature. 2008; 453: 921–924. 10.1038/nature06975 18488016 PMC2652570

[5] Kocerha J, Liu Y, Willoughby D, Chidamparam K, Benito J, Nelson K, et al Longitudinal transcriptomic dysregulation in the peripheral blood of transgenic Huntington's disease monkeys. BMC Neurosci. 2013; 14: 88 10.1186/1471-2202-14-88 23957861 PMC3751855

[6] Aylward EH, Liu D, Nopoulos PC, Ross CA, Pierson RK, Mills JA, et al Striatal volume contributes to the prediction of onset of Huntington disease in incident cases. Biol Psychiatry. 2012; 71: 822–828. 10.1016/j.biopsych.2011.07.030 21907324 PMC3237730

[7] Paulsen JS, Nopoulos PC, Aylward E, Ross CA, Johnson H, Magnotta VA, et al Striatal and white matter predictors of estimated diagnosis for Huntington disease. Brain Res Bull. 2010; 82: 201–207. 10.1016/j.brainresbull.2010.04.003 20385209 PMC2892238

[8] Tabrizi SJ, Scahill RI, Durr A, Roos RA, Leavitt BR, Jones R, et al Biological and clinical changes in premanifest and early stage Huntington's disease in the TRACK-HD study: the 12-month longitudinal analysis. Lancet Neurol. 2011; 10: 31–42. 10.1016/S1474-4422(10)70276-3 21130037

[9] Tabrizi SJ, Scahill RI, Owen G, Durr A, Leavitt BR, Roos RA, et al Predictors of phenotypic progression and disease onset in premanifest and early-stage Huntington's disease in the TRACK-HD study: analysis of 36-month observational data. Lancet Neurol. 2013; 12: 637–649. 10.1016/S1474-4422(13)70088-7 23664844

[10] Putkhao K, Kocerha J, Cho IK, Yang J, Parnpai R, Chan AWS. Pathogenic cellular phenotypes are germline transmissible in a transgenic primate model of Huntington's disease. Stem Cells Dev. 2013; 22: 1198–1205. 10.1089/scd.2012.0469 23190281 PMC3613972

[11] Groen JL, de Bie RM, Foncke EM, Roos RA, Leenders KL, Tijssen MA. Late-onset Huntington disease with intermediate CAG repeats: true or false? J Neurol Neurosurg Psychiatry. 2010; 81: 228–230. 10.1136/jnnp.2008.170902 20145031

[12] Rubinsztein DC, Leggo J, Coles R, Almqvist E, Biancalana V, Cassiman JJ, et al Phenotypic characterization of individuals with 30–40 CAG repeats in the Huntington disease (HD) gene reveals HD cases with 36 repeats and apparently normal elderly individuals with 36–39 repeats. Am J Hum Genet. 1996; 59: 16–22. 8659522 PMC1915122

[13] Squitieri F, Jankovic J. Huntington's disease: how intermediate are intermediate repeat lengths? Mov Disord. 2012; 27: 1714–1717. 10.1002/mds.25172 23008174

[14] Padowski JM, Weaver KE, Richards TL, Laurino MY, Samii A, Aylward EH, et al Neurochemical correlates of caudate atrophy in Huntington's disease. Mov Disord. 2014; 29: 327–335. 10.1002/mds.25801 24442623 PMC3960319

[15] Unschuld PG, Edden RA, Carass A, Liu X, Shanahan M, Wang X, et al Brain metabolite alterations and cognitive dysfunction in early Huntington's disease. Mov Disord. 2012; 27: 895–902. 10.1002/mds.25010 22649062 PMC3383395

[16] Mittoux V, Joseph JM, Conde F, Palfi S, Dautry C, Poyot T, et al Restoration of cognitive and motor functions by ciliary neurotrophic factor in a primate model of Huntington's disease. Hum Gene Ther. 2000; 11: 1177–1187. 10834619 10.1089/10430340050015220

[17] Palfi S, Conde F, Riche D, Brouillet E, Dautry C, Mittoux V, et al Fetal striatal allografts reverse cognitive deficits in a primate model of Huntington disease. Nat Med. 1998; 4: 963–966. 9701252 10.1038/nm0898-963

[18] Palfi S, Ferrante RJ, Brouillet E, Beal MF, Dolan R, Guyot MC, et al Chronic 3-nitropropionic acid treatment in baboons replicates the cognitive and motor deficits of Huntington's disease. J Neurosci. 1996; 16: 3019–3025. 8622131 10.1523/JNEUROSCI.16-09-03019.1996 PMC6579050

[19] Roitberg BZ, Emborg ME, Sramek JG, Palfi S, Kordower JH. Behavioral and morphological comparison of two nonhuman primate models of Huntington's disease. Neurosurgery. 2002; 50: 137–145; discussion 145–136. 11844244 10.1097/00006123-200201000-00022

[20] Taylor JR, Elsworth JD, Roth RH, Sladek JR Jr., Redmond DE Jr. Cognitive and motor deficits in the acquisition of an object retrieval/detour task in MPTP-treated monkeys. Brain. 1990; 113 (Pt 3): 617–637. 2364263 10.1093/brain/113.3.617

[21] McBride JL, Pitzer MR, Boudreau RL, Dufour B, Hobbs T, Ojeda SR, et al Preclinical safety of RNAi-mediated HTT suppression in the rhesus macaque as a potential therapy for Huntington's disease. Mol Ther. 2011; 19: 2152–2162. 10.1038/mt.2011.219 22031240 PMC3242667

[22] Bachevalier J, Landis LS, Walker LC, Brickson M, Mishkin M, Price DL, et al Aged monkeys exhibit behavioral deficits indicative of widespread cerebral dysfunction. Neurobiol Aging. 1991; 12: 99–111. 2052134 10.1016/0197-4580(91)90048-o

[23] Darling WG, Peterson CR, Herrick JL, McNeal DW, Stilwell-Morecraft KS, Morecraft RJ. Measurement of coordination of object manipulation in non-human primates. J Neurosci Methods.2006; 154: 38–44. 16464505 10.1016/j.jneumeth.2005.11.013

[24] Lacreuse A, Diehl MM, Goh MY, Hall MJ, Volk AM, Chhabra RK, et al Sex differences in age-related motor slowing in the rhesus monkey: behavioral and neuroimaging data. Neurobiol Aging. 2005; 26: 543–551. 15653182 10.1016/j.neurobiolaging.2004.05.007

[25] Zhang Z, Andersen A, Smith C, Grondin R, Gerhardt G, Gash D. Motor slowing and parkinsonian signs in aging rhesus monkeys mirror human aging. J Gerontol A Biol Sci Med Sci. 2000; 55: B473–480. 11034220 10.1093/gerona/55.10.b473

[26] Biglan K, Shoulson I. Juvenile-onset huntington disease: a matter of perspective. Arch Neurol. 2007; 64: 783–784. 17562925 10.1001/archneur.64.6.783

[27] Cloud LJ, Rosenblatt A, Margolis RL, Ross CA, Pillai JA, Corey-Bloom J, Seizures in juvenile Huntington's disease: frequency and characterization in a multicenter cohort. Mov Disord. 2012; 27: 1797–1800. 10.1002/mds.25237 23124580

[28] Geevasinga N, Richards FH, Jones KJ, Ryan MM. Juvenile Huntington disease. J Paediatr Child Health. 2006; 42: 552–554. 16925544 10.1111/j.1440-1754.2006.00921.x

[29] Squitieri F, Frati L, Ciarmiello A, Lastoria S, Quarrell O. Juvenile Huntington's disease: does a dosage-effect pathogenic mechanism differ from the classical adult disease? Mech Ageing Dev. 2006; 127: 208–212. 16274727 10.1016/j.mad.2005.09.012

[30] Paulsen JS, Smith MM, Long JD. Cognitive decline in prodromal Huntington Disease: implications for clinical trials. J Neurol Neurosurg Psychiatry. 2013; 84: 1233–1239. 10.1136/jnnp-2013-305114 23911948 PMC3795884

[31] Courtine G, Bunge MB, Fawcett JW, Grossman RG, Kaas JH, Lemon R, et al Can experiments in nonhuman primates expedite the translation of treatments for spinal cord injury in humans? Nat Med. 2007; 13: 561–566. 17479102 10.1038/nm1595 PMC3245971

[32] Bachevalier J, Machado CJ, Kazama A. Behavioral outcomes of late-onset or early-onset orbital frontal cortex (areas 11/13) lesions in rhesus monkeys. Ann N Y Acad Sci. 2011; 1239: 71–86. 10.1111/j.1749-6632.2011.06211.x 22145877 PMC3740330

[33] Ewing-Cobbs L, Prasad MR, Swank P, Kramer L, Mendez D, Treble A, et al Social communication in young children with traumatic brain injury: Relations with corpus callosum morphometry. Int J Dev Neurosci. 2012; 30: 247–254. 10.1016/j.ijdevneu.2011.07.004 21807088 PMC3265631

[34] Rice J. Animal models: Not close enough. Nature. 2012; 484: S9–S9. 22509510 10.1038/nature11102

[35] Aziz NA, Pijl H, Frolich M, Snel M, Streefland TC, Roelfsema F, et al Systemic energy homeostasis in Huntington's disease patients. J Neurol Neurosurg Psychiatry. 2010; 81: 1233–1237. 10.1136/jnnp.2009.191833 20710011

[36] Gaba AM, Zhang K, Marder K, Moskowitz CB, Werner P, Boozer CN. Energy balance in early-stage Huntington disease. Am J Clin Nutr. 2005; 81: 1335–1341. 15941884 10.1093/ajcn/81.6.1335

[37] Hamilton JM, Wolfson T, Peavy GM, Jacobson MW, Corey-Bloom J. Rate and correlates of weight change in Huntington's disease. J Neurol Neurosurg Psychiatry. 2004; 75: 209–212. 14742590 10.1136/jnnp.2003.017822 PMC1738924

[38] Sackett GP, Ruppenthal GC, Davis AE. Survival, growth, health, and reproduction following nursery rearing compared with mother rearing in pigtailed monkeys (Macaca nemestrina). Am J Primatol. 2002; 56: 165–183. 11857653 10.1002/ajp.1072

[39] Goursaud AP, Bachevalier J. Social attachment in juvenile monkeys with neonatal lesion of the hippocampus, amygdala and orbital frontal cortex. Behav Brain Res. 2007; 176: 75–93. 17084912 10.1016/j.bbr.2006.09.020

[40] Li CX, Patel S, Auerbach EJ, Zhang X. Dose-dependent effect of isoflurane on regional cerebral blood flow in anesthetized macaque monkeys. Neurosci Lett. 2013; 541: 58–62. 10.1016/j.neulet.2013.02.007 23428509 PMC4349366

[41] Smith SM, Jenkinson M, Woolrich MW, Beckmann CF, Behrens TE, Johansen-Berg H, et al Advances in functional and structural MR image analysis and implementation as FSL. Neuroimage. 2004; 23 Suppl 1: S208–219. 15501092 10.1016/j.neuroimage.2004.07.051

[42] Adluru N, Zhang H, Fox AS, Shelton SE, Ennis CM, Bartosic AM, et al A diffusion tensor brain template for rhesus macaques. Neuroimage. 2012; 59: 306–318. 10.1016/j.neuroimage.2011.07.029 21803162 PMC3195880

[43] Provencher SW. Estimation of metabolite concentrations from localized in vivo proton NMR spectra. Magn Reson Med. 1993; 30: 672–679. 8139448 10.1002/mrm.1910300604

[44] Haga KK, Khor YP, Farrall A, Wardlaw JM. A systematic review of brain metabolite changes, measured with 1H magnetic resonance spectroscopy, in healthy aging. Neurobiol Aging. 2009; 30: 353–363. 17719145 10.1016/j.neurobiolaging.2007.07.005

[45] Herndon JG, Constantinidis I, Moss MB. Age-related brain changes in rhesus monkeys: a magnetic resonance spectroscopic study. Neuroreport. 1998; 9: 2127–2130. 9674606 10.1097/00001756-199806220-00040

[46] Gundersen HJ. Stereology of arbitrary particles. A review of unbiased number and size estimators and the presentation of some new ones, in memory of William R. Thompson. J Microsc. 1986; 143: 3–45. 3761363

