# Supplementary figures and images for: Progressive Cognitive Deficit, Motor Impairment and Striatal Pathology in a Transgenic Huntington Disease Monkey Model from Infancy to Adulthood
Source: PLoS One. 2015 May 12;10(5):e0122335. doi: 10.1371/journal.pone.0122335 (PMC4428630; doi:10.1371/journal.pone.0122335)

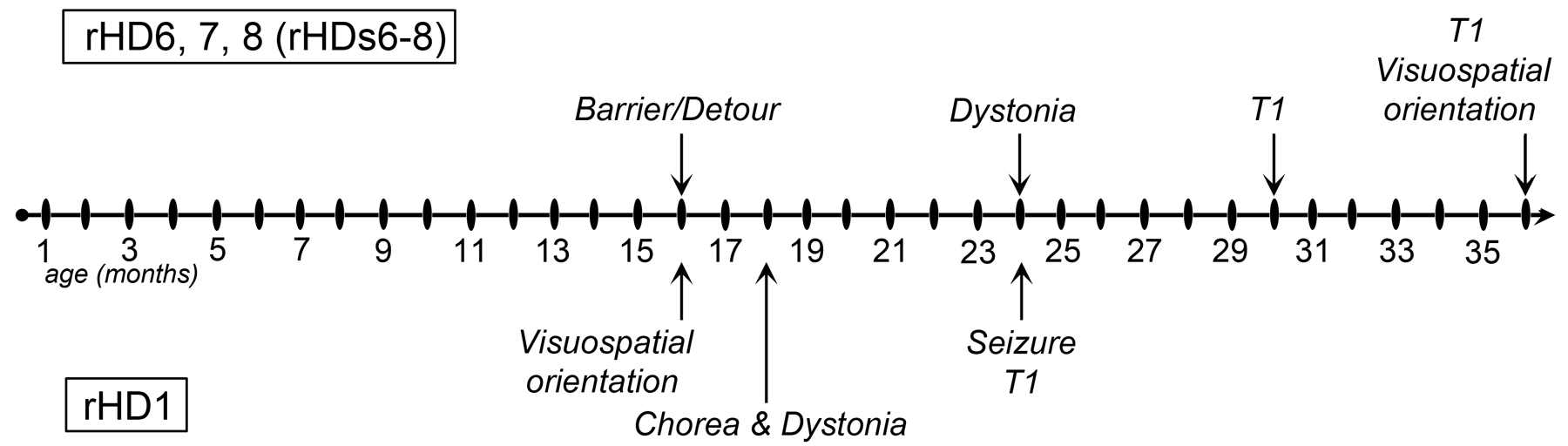

Supplement: S1 Fig — Specific types of impairment, including cognitive motor functions and neuroanatomical changes, are plotted on a timeline for rHDs6-8 (top) and rHD1 (bottom). (TIF) [file pone.0122335.s001.tif]

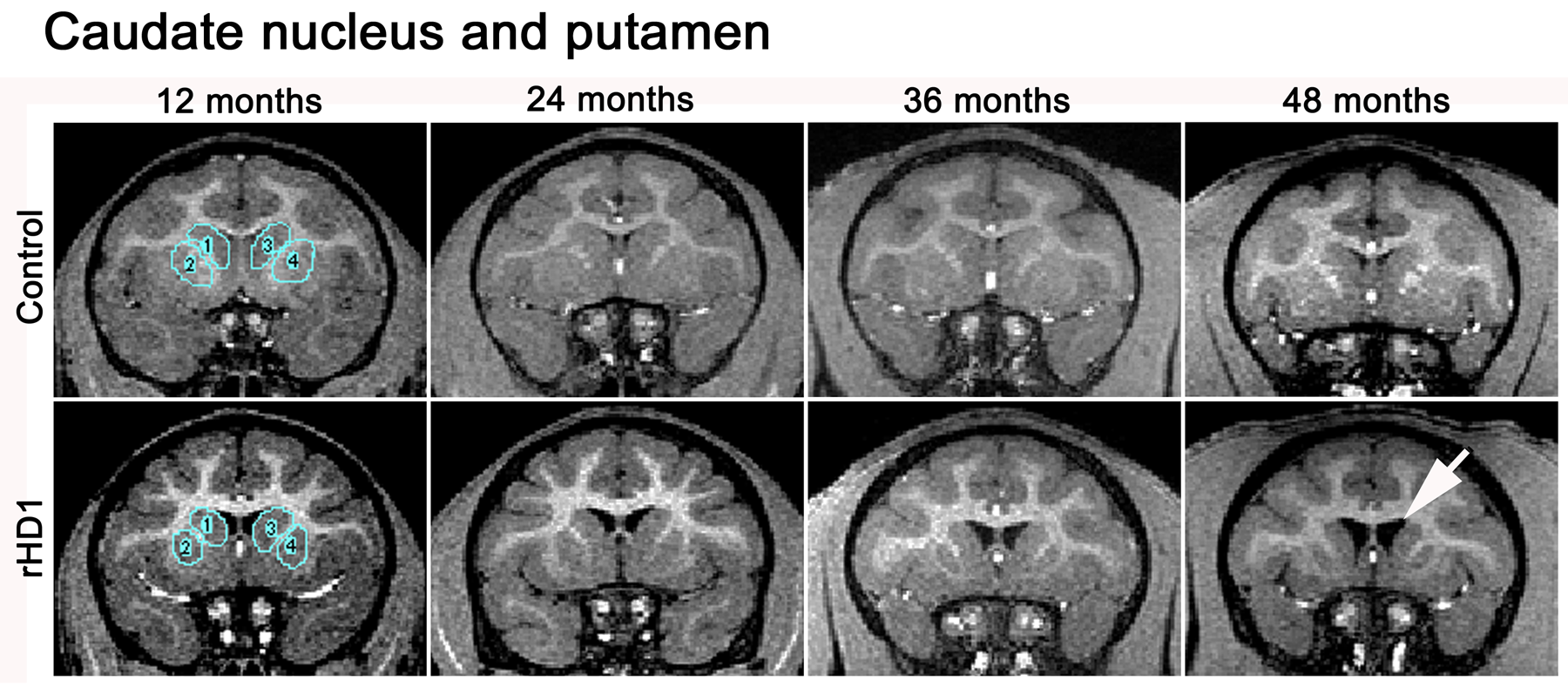

Supplement: S2 Fig — (TIF) [file pone.0122335.s002.tif]

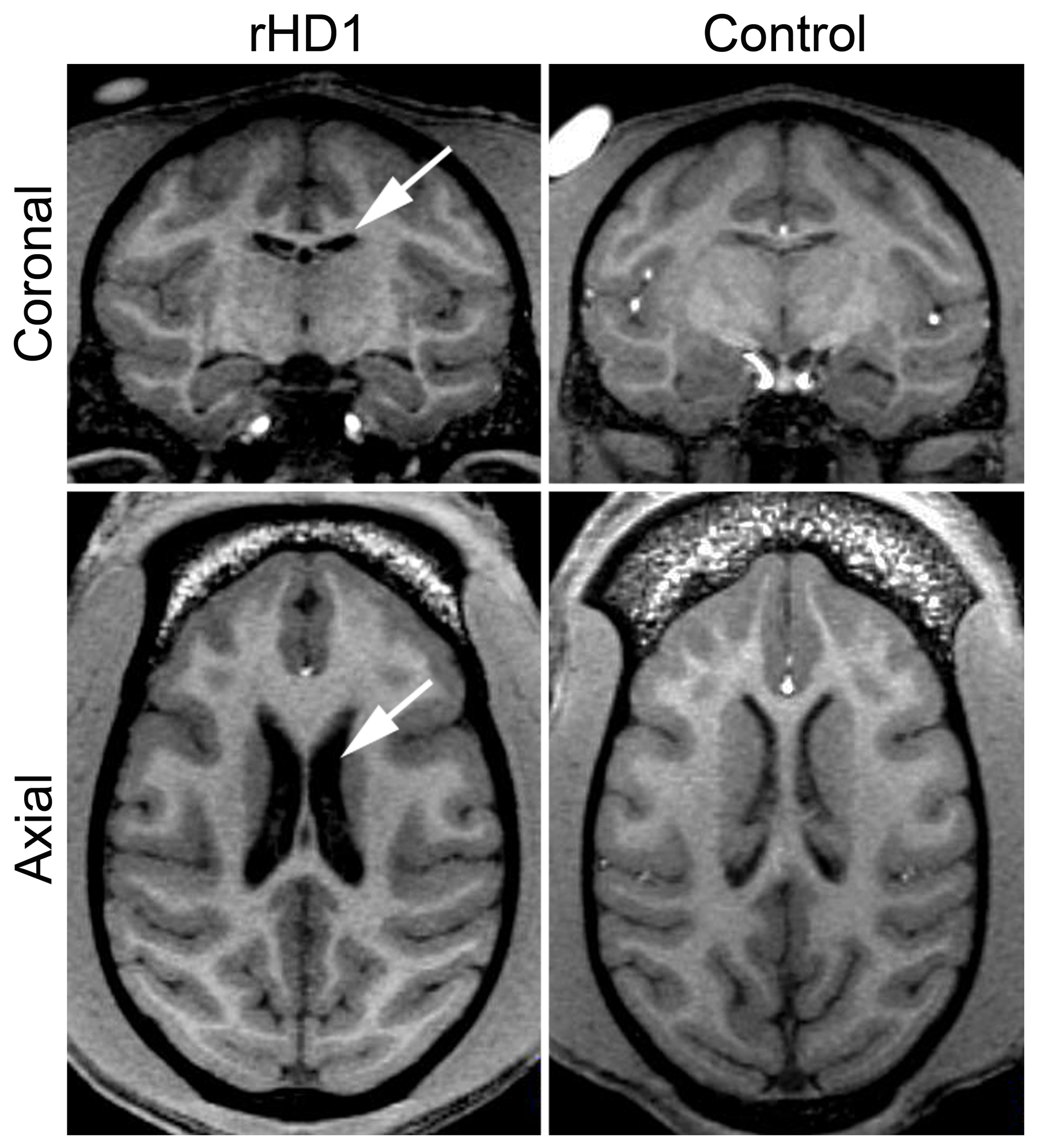

Supplement: S3 Fig — Arrows indicate enlarged ventricle. (TIF) [file pone.0122335.s003.tif]

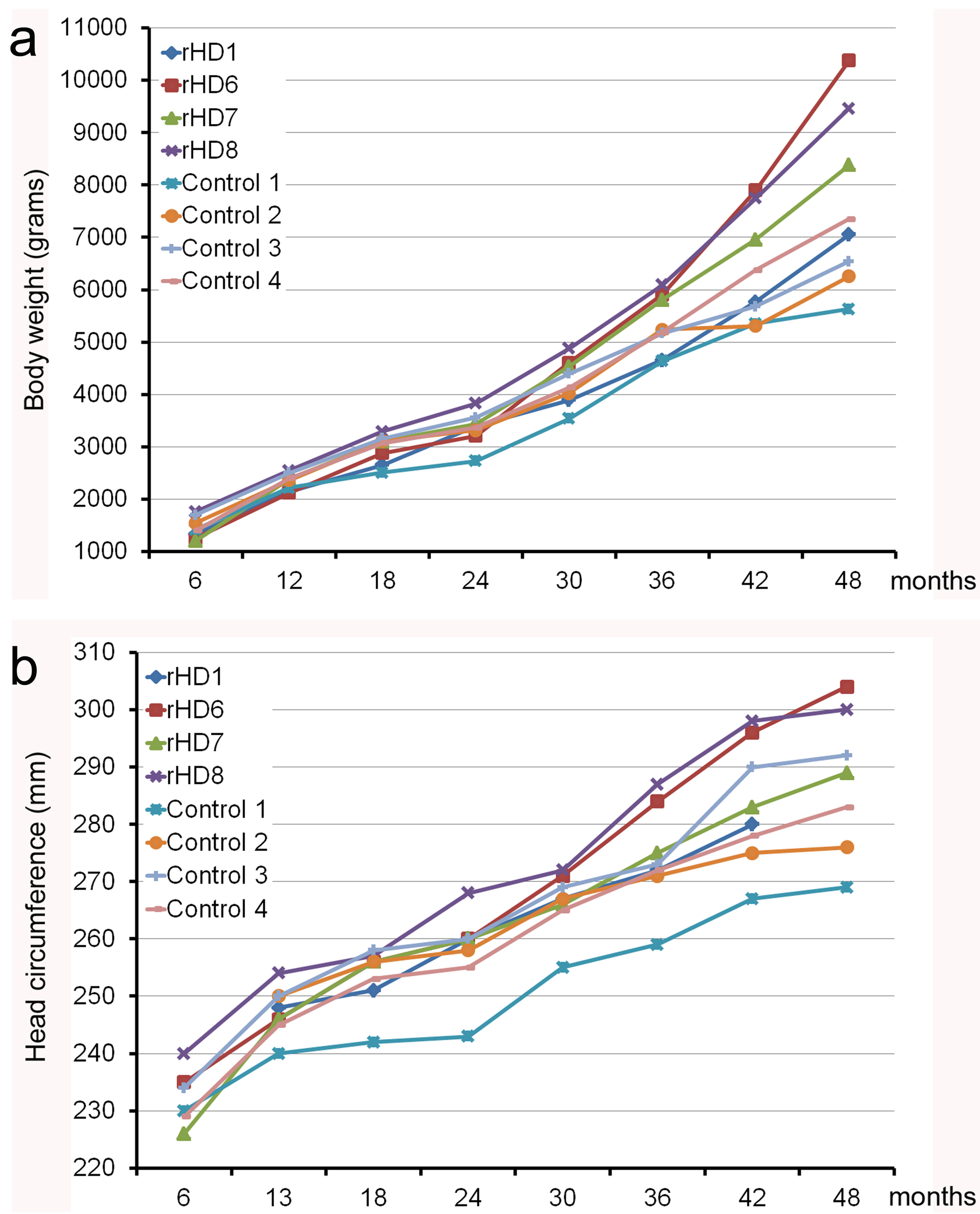

Supplement: S4 Fig — Controls 1 and 2 are female monkeys. Controls 3 and 4 are male monkeys. (TIF) [file pone.0122335.s004.tif]
