# Supplementary material for: Progressive Cognitive Deficit, Motor Impairment and Striatal Pathology in a Transgenic Huntington Disease Monkey Model from Infancy to Adulthood
Source: PLoS One. 2015 May 12;10(5):e0122335. doi: 10.1371/journal.pone.0122335 (PMC4428630; doi:10.1371/journal.pone.0122335)
Supplement: S1 Table — (DOCX) [file pone.0122335.s005.docx]

**Table S1. Definitions of behavioral measures in the Object Retrieval Detour Task (ORDT)**

| **Measures** | **Definitions** |
| --- | --- |
| Response initiation latency | Time from raising the screen at the start of the trial until the subject  makes contact with the test box or reward |
| Correct reaches | Retrieval of the reward on a trial |
| Success reaches | Retrieval of the reward on the first reach of the trial |
| Unsuccessful reaches | Reaching into the open side of the box but failing to retrieve the reward or dropping it |
| Barrier reaches | Reaching to the closed, transparent side of the box |
| Total reaches | Number of reaches on a trial |
| Perseverative reaches | A trial in which the first reach was made into the barrier and is repeated over and over. These reaches were not included in “Total reaches” |
