# Supplementary material for: Progressive Cognitive Deficit, Motor Impairment and Striatal Pathology in a Transgenic Huntington Disease Monkey Model from Infancy to Adulthood
Source: PLoS One. 2015 May 12;10(5):e0122335. doi: 10.1371/journal.pone.0122335 (PMC4428630; doi:10.1371/journal.pone.0122335)
Supplement: S2 Table — (DOCX) [file pone.0122335.s006.docx]

**Table S2. List of primers**

| **Gene of Interest** | **Forward (5’🡪3’)** | **Reverse (5’🡪3’)** | **Amplicon Size** | **[Final]_reaction_** |
| --- | --- | --- | --- | --- |
| *Ubc* (rhesus)  Acc. No. XM_001102090.2 | 5’-CCA CTC TGC ACT TGG TCC TG-3’ | 5’-CCA GTT GGG AAT GCA ACA ACT TTA-3’ | 113 bp | 0.4 μM |
| *Htt*, exon 1 (rhesus) XM_001086119.2 | 5’-GCG ACC CTG GAA AAG CTG AT-3’ | 5’- CTG CTG CTG CTG GAA GGA CT-3’ | 60 bp | 0.4 μM |
| *Htt*, exon 26 (rhesus) XM_001086119.2 | 5’-ACC CTG CTC TCG TCA GCT TGG-3’ | 5’-AGC AAG TTT CCG GCC AAA AT-3’ | 60 bp | 0.4 μM |
